# Evaluating a multifaceted stewardship intervention on proton pump inhibitor utilization: an interrupted time-series analysis of prescribing patterns in a northwest Chinese hospital

**DOI:** 10.3389/fphar.2026.1700146

**Published:** 2026-02-10

**Authors:** Xiaoling Wang, David J. McIver, Hui Min, Jia Guo, Haiyan Li

**Affiliations:** 1 Department of Gastroenterology, Xi’an People’s Hospital (Xi’an Fourth Hospital), Xi’an, China; 2 Mclver Epi Scientific Consulting, Nanaimo, BC, Canada; 3 Department of Pharmacy, Xi’an People’s Hospital (Xi’an Fourth Hospital), Xi’an, China

**Keywords:** clinical pharmacists, hospital, interrupted time series, national guidelines, national key monitoring drugs, proton pump inhibitors

## Abstract

**Background:**

Promoting the rational use of proton pump inhibitors (PPIs) has become an important aspect in controlling the growth of pharmaceutical expenditures in China. In recent years, the first national guidelines for the clinical application of PPIs and the second batch of the China National Key Monitoring Drug (NKMD) policy were released to regulate PPI utilization. However, few studies have fully investigated the impact of the multifaceted stewardship intervention on PPI utilization in hospitals, especially in northwestern China.

**Methods:**

We collected monthly PPI usage data over 8 years from a tertiary hospital of northwestern China between January 2017 and December 2024. Multi-intervention interrupted time-series (ITS) analysis was used to evaluate the change in the defined daily dose (DDD) per 100 bed-days under the intervention measures managed by clinical pharmacists through the multifaceted stewardship.

**Results:**

Although the DDD per 100 bed-days for overall PPIs decreased by 12.93 (*p* = 0.009) under the national guidelines (intervention_1_), no significant time trend in the overall PPI utilization was observed (*p* = 0.603). However, following the implementation of the NKMD policy (intervention_2_), a significant decreasing trend (β_4_ = −0.46, *p* = 0.011) emerged in the DDD per 100 bed-days for overall PPIs. The national guidelines (intervention_1_) led to significant immediate reductions in both oral (β_1_ = −5.67, *p* = 0.005) PPI consumption and intravenous (β_1_ = −7.26, *p* = 0.020) PPI consumption. However, the trend for oral PPIs subsequently exhibited a significant upward slope (β_2_ = 0.26, *p* = 0.015) under the national guidelines (intervention_1_). Conversely, the consumption of oral PPIs showed a significant sustained linear decreasing trend (β_4_ = −0.58, *p* < 0.001) under the NKMD policy (intervention_2_).

**Conclusion:**

The implementation of the national guidelines led to a significant immediate reduction, but no significant time trend in the overall PPI utilization was observed. Our findings highlight the impact of the NKMD policy on the sustained downward trend in overall and oral PPI consumption. The prospective prescription review system may be effective in promoting the long-term rational use of PPIs in clinical practice.

## Introduction

Proton pump inhibitors (PPIs) are a class of drugs suppressing gastric acid secretion, which are extensively used for acid-related disorders. PPIs are among the most widely prescribed agents worldwide (Forgacs and Loganayagam, 2008). Inappropriate PPI prescribing—such as lack of indication, inappropriate drug selection, dosage, administration route, treatment duration, solvent choice, or solvent volume—has been widely reported in China and other countries, potentially increasing healthcare costs and the risk of adverse outcomes (Lassalle et al., 2020; Liu et al., 2020; Sattayalertyanyong et al., 2020; Ying et al., 2019). Approximately 10% of people in Spain took PPIs daily in 2016, and PPIs accounted for 7.4% and 3.4% of total packages and total national pharmaceutical expenditures, respectively (Torres-Bondia et al., 2022). In Switzerland, the consumption of PPIs increased from 19.7% in 2012 to 23.0% in 2017, and inappropriate PPI prescriptions increased from 4.8% in 2013 to 6.4% in 2017 (Muheim et al., 2021). In China, PPI use has markedly increased over the past two decades (Luo et al., 2018). An appreciable 10.4-fold increase in consumption was observed between 2004 and 2013 at the largest teaching hospital in Chongqing (Zeng et al., 2015). In China, 32.6%–56.8% of PPI prescriptions were for inappropriate indications (Ying et al., 2019). The annual consumption of PPIs in China exceeds that of both antihypertensive and antidiabetic drugs, ranking second only to antibacterial agents (Hong et al., 2020). This excessive use of PPIs not only increases the risk of various adverse reactions but also imposes a substantial financial burden on patients (Zhang et al., 2021; Zhang et al., 2025).

To promote the rationality of PPI use, the Guidelines for Clinical Application of Proton Pump Inhibitors were released by the National Health Commission of the People’s Republic of China in December 2020 (National Health Commission of the People’s Republic of China, 2020). This highlights that the appropriate use of PPIs in clinical practice has been elevated to the national level. The issued national guidelines provide recommendations on indications, dosage, administration routes, and treatment duration for PPIs. The national guidelines also encompass regulations for clinical application and prescription stewardship measures. Serving as the guiding principles, the national guidelines have become the basis for standardizing the prescribing behavior of doctors.

In general, drugs with high prices, large consumption, and unclear therapeutic effects are identified as key monitoring drugs (Li et al., 2022b). The establishment of key monitoring drugs is to reduce the irrational clinical use of drugs in the catalog and control the unreasonable growth of pharmaceutical expenditures (Wen et al., 2023). The first and second batches of the catalog of National Key Monitoring Drug (NKMD) policy were released in July 2019 and January 2023, and a total of 20 and 30 drugs were included, respectively. A total of five types of PPIs (omeprazole, esomeprazole, pantoprazole, lansoprazole, and rabeprazole) were included in the second batch of the catalog. Previous studies have reported that the implementation of the first batch of the NKMD policy catalog led to a significant and sustained decrease in the consumption of policy-related drugs (Li et al., 2022b; Wen et al., 2023).

The national guidelines and the NKMD policy were associated with a decrease in the consumption of policy-related drugs (Li et al., 2022b; Wen et al., 2023; Ziegler et al., 2019). So far, the impact of the multifaceted stewardship interventions on PPI utilization remains unclear in China. We hypothesized that the national guidelines and the NKMD policy may lead to a decrease in the consumption of PPIs. To this end, we conducted the present study to investigate the short- and long-term effects of multifaceted stewardship interventions on PPI utilization in a large tertiary hospital in northwestern China.

## Materials and methods

### Study setting

In this study, monthly data on the consumption of PPIs from January 2017 to December 2024 were obtained from the hospital information system (HIS) of Xi’an People’s Hospital (Xi’an Fourth Hospital). This tertiary hospital is located in Shaanxi Province, northwestern China, covers two districts, and has approximately 1,300 beds. This medical institution, which offers comprehensive medical, academic, and scientific research capacities, is capable of an average daily admission rate of approximately 6,743 patients and >145,300 inpatient admissions annually.

### Study design

We used a multi-intervention interrupted time-series (ITS) analysis to evaluate the impact of the national guidelines and the NKMD policy on monthly PPI utilization. The study spanned from January 2017 to December 2024, divided into three periods: 1) the pre-policy period (January 2017–December 2020), 2) the post-policy_1_ period (January 2021–December 2022), following the release of the national guideline, and 3) the post-policy_2_ period (January 2023–December 2024), after the implementation of the NKMD policy. This design aligns with methodological recommendations for segmented regression in ITS analysis, which typically require a minimum of 10–12 time points per segment to reliably estimate segment-specific slopes and breakpoint effects and to reduce bias from autocorrelation.

### Intervention measures

Intervention measures developed through the implementation of the multifaceted stewardship included 1) the establishment of a management group, 2) educational programs, 3) prescription evaluation and audits, 4) cooperation with the information department, and 5) administrative interventions. The intervention measures were implemented constantly by clinical pharmacists at the study hospital from January 2021 to December 2024. The detailed intervention strategies are further described in the Supplementary Material (see Supplement 1).

### Data collection

Six brand and generic PPIs were available for analysis, namely, esomeprazole (oral and injectable), omeprazole (oral and injectable), lansoprazole (oral and injectable), pantoprazole (oral and injectable), rabeprazole (oral), and ilaprazole (injectable), in our hospital throughout the study period. The following PPI utilization data were collected based on monthly aggregated estimates: generic name, dosage form, specification, pharmaceutical manufacturer, and purchase volume. The consumption data were captured and extracted from the HIS when PPIs were prescribed. The consumption data for each PPI preparation were collected on a monthly and individual basis, facilitating the elimination of recall bias or misclassification bias.

A retrospective pre- and post-policy study was conducted. Patients (≥18 years) who underwent surgery for fractures and were prescribed PPIs were enrolled from the orthopedic department to assess the appropriateness of stress ulcer prophylaxis (SUP). Data were collected for three periods: pre-policy (January 2017–December 2020), post-policy_1_ (January 2021–December 2022), and post-policy_2_ (January 2023–December 2024). Electronic medical records were reviewed, and sociodemographic information was collected during each period. Sociodemographic characteristics included age, gender, occupational status, and place of residence. The following clinical data were extracted from the electronic medical records: diagnosis at admission, comorbidities, length of hospital stay, surgical details (including operation time), and PPI usage (generic name, dosage, timing of the first prophylaxis dose, indications, and duration). The appropriateness of SUP was evaluated based on the national guidelines.

### Outcome indicators

The defined daily dose (DDD) per 100 bed-days was used to assess the impact of multifaceted stewardship interventions on PPI consumption trends.

The consumption of PPIs among inpatients was converted to DDD per 100 bed-days to enable comparisons (WHO Collaborating Centre for Drug Statistics Methodology, 2022a). According to the Anatomical and Therapeutic Classification (ATC) code A02BC, the recommended DDDs of omeprazole, rabeprazole, lansoprazole, esomeprazole, pantoprazole, and ilaprazole are 20 mg, 20 mg, 30 mg, 30 mg, 40 mg, and 10 mg, respectively (WHO Collaborating Centre for Drug Statistics Methodology, 2022b). The DDD was calculated as total PPI consumption/DDD. The total DDDs for each month were calculated by summing the DDDs of each PPI preparation. The higher the DDD value, the higher the frequency of the drug use. A bed-day was a day during which a person was confined to a bed and stayed overnight in the hospital (WHO Collaborating Centre for Drug Statistics Methodology, 2022a).

Four outcomes were compared before and after the implementation of clinical pharmacist-managed interventions to assess the appropriateness of prescription patterns of SUP: (1) the incidence of inappropriate indications for SUP use, (2) the average percentage of patient-days with inappropriate SUP use, (3) the average duration of inappropriate SUP use, and (4) the rate of inappropriate timing of the first prophylactic dose (defined as administration not prior to surgery when SUP was indicated).

According to the national guidelines, SUP is recommended only for intensive care unit (ICU) patients or those with high-risk factors. Based on this, SUP indications were assessed as follows: patients who received PPI prescriptions and had risk factors were classified as having appropriate indications, whereas those who received PPI prescriptions but had no risk factors were classified as having inappropriate indications. The average percentage of patient-days of inappropriate SUP use was calculated by dividing the total inappropriate PPI patient-days by the total patient-days of PPI use. We selected this patient-day metric, rather than the number of patients, to more accurately quantify the extent of inappropriate use and to minimize the confounding effect of variation in the duration of therapy. According to the national guidelines, PPIs were recommended to be prescribed prior to surgery when SUP was indicated.

### Statistical analysis

First, ITS analysis was used to estimate the effects of the policies. The regression model was constructed as follows:

$$Y=\alpha +\beta_{0}*\text{time}+\beta_{1}*{\text{intervention}}_{1}+\beta_{2}*\text{time}*{\text{intervention}}_{1}+\beta_{3}*{\text{intervention}}_{2}+\beta_{4}*\text{time}*{\text{intervention}}_{2}+\beta_{5}*\text{COVID}-19+\beta_{6}*\text{Month}+\varepsilon_{\text{it}},$$

where Y refers to the outcome variables. Time is a continuous variable of observation months. For the DDD per 100 bed-days, intervention_1_ and intervention_2_ are the dummy variables of the policy intervention time. Intervention_1_ was coded 1 under the implementation of the national guidelines (January 2021 to December 2024), otherwise coded 0. Intervention_2_ was coded 1 under the implementation of the NKMD policy (January 2023 to December 2024), otherwise coded 0. α and β_0_ represent the intercept and slope of the outcome in the pre-intervention period of the policies, respectively. β_1_ and β_3_ represent the immediate level change in dependent variables at the point of implementing the intervention. β_2_ and β_4_ represent the slope change in dependent variables during intervention periods. The COVID-19 pandemic was defined as 1 for the period from January 2020 to April 2020. Month is a covariate variable to adjust the monthly trend. The effect of the outbreak of the COVID-19 pandemic is considered β_5_, and the month effect is considered β_6_. ε_it_ is an estimate of the random error.

Newey–West corrections were applied to account for autocorrelation in the model residuals. A sensitivity analysis was carried out to validate the robustness of the results. Considering the time required for these policies to take effect, we set a 1-month lag to evaluate the stability of policy effects. Data were managed and analyzed in R 4.4.0. A difference with a *p*-value <0.05 was considered statistically significant. Full model diagnostics is provided in the Supplementary Material (see Supplement 2).

Second, descriptive statistics were used to present the survey data of the prescription patterns of SUP. Categorical variables were presented as counts and percentages and continuous variables as means and standard deviations (SD). Differences in the variables among the pre-policy, post-policy_1_, and post-policy_2_ groups were evaluated using the Chi-square test for categorical variables and using the independent samples t-test for normal continuous variables. Statistical analysis was performed using SPSS Statistics version 25. A ***p*-**value <0.05 was considered statistically significant.

## Results

### Changes in the DDD per 100 bed-days for PPIs during different periods

**FIGURE 1:**
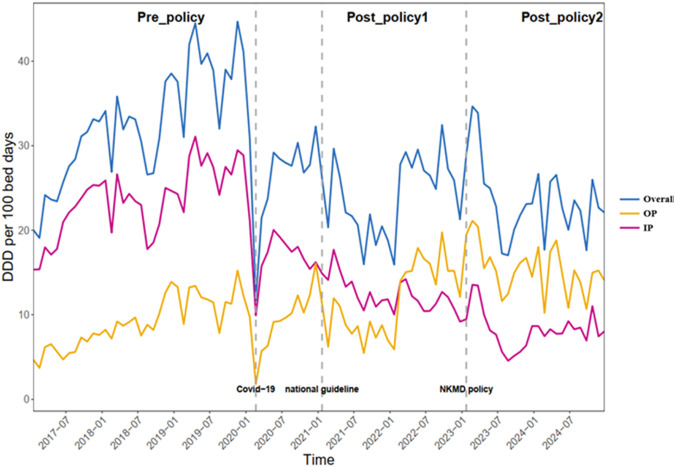
Changes in DDDs per 100 bed-days for all the PPIs during different periods.

Changes in the DDD per 100 bed-days for PPIs were observed and recorded from January 2017 to December 2024, which was divided into three periods. Figure 1 demonstrates the temporal changes in DDD per 100 bed-days for overall PPIs, the oral preparations (OPs) combined, and the intravenous preparations (IPs) combined across three periods: pre-policy (48 months), post-policy_1_ (24 months), and post-policy_2_ (24 months).

During the pre-policy period (January 2017 to December 2020), IP combined accounted for the majority of the overall utilization (ranged from 50.2% to 84.7%) and OP combined remained at a relatively low level (mean 29.0%). A sharp decrease in DDD per 100 bed-days of PPIs was observed around February 2020, which aligned with the lockdown in China during the outbreak of the COVID-19 pandemic. During the post-policy_1_ period (January 2021 to December 2022), the consumption of overall PPIs rebounded but remained lower than the peaks during the pre-policy period. The DDD per 100 bed-days of OP combined was consistently higher than IP combined after February 2022. During the post-policy_2_ period (January 2023 to December 2024), following the NKMD policy released in January 2023, the DDDs of PPIs at the end of 2024 showed a lower level than those at the beginning of 2023.

### Changes in DDDs per 100 bed-days for overall PPIs under the multifaceted stewardship

**FIGURE 2:**
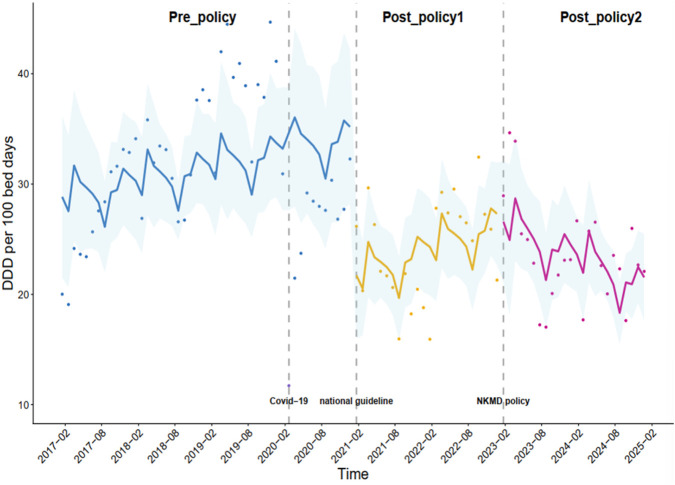
Monthly DDDs per 100 bed-days for all the PPIs combined.

As shown in Table 1, although the DDD per 100 bed-days for overall PPIs decreased by 12.93 (*p* = 0.009) under the national guidelines (intervention_1_), no significant time trend in the overall PPI utilization was observed (*p* = 0.603). The DDD per 100 bed-days for overall PPIs substantially decreased by 20.19 because of the outbreak of the COVID-19 pandemic (*p* < 0.001). However, a significant decreasing trend (β_4_ = −0.46, *p* = 0.011) in the DDD per 100 bed-days for overall PPIs was observed under the implementation of the NKMD policy (intervention_2_) (Figure 2).

**TABLE 1:** Interrupted time-series analyses of DDDs per 100 bed-days for overall PPIs.

Category	Newey–West coefficient	95% CI	*p*-value
Pre-intervention trend (β_0_)	0.12	−0.13 to 0.37	0.331
Change in the level for intervention_1_ (β_1_)	−12.93	−22.54 to −3.32	**0.009**
Change in the trend for intervention_1_ (β_2_)	0.09	−0.26 to 0.45	0.603
Change in the level for intervention_2_ (β_3_)	−0.28	−8.04 to 7.48	0.943
Change in the trend for intervention_2_ (β_4_)	−0.46	−0.82 to −0.11	**0.011**
COVID-19 (β_5_)	−20.19	−26.62 to −13.75	**<0.001**
Constant	28.84	21.51–36.16	**<0.001**

Bold values indicate a *p*-value <0.05.

The implementation of the national guidelines (intervention_1_) resulted in a significant immediate reduction, but no significant time trend in the overall PPI utilization was observed. A significant decreasing trend was observed in the DDD per 100 bed-days for overall PPIs under the implementation of the NKMD policy (intervention_2_).

### Changes in the DDD per 100 bed-days for oral PPIs under the multifaceted stewardship

**FIGURE 3:**
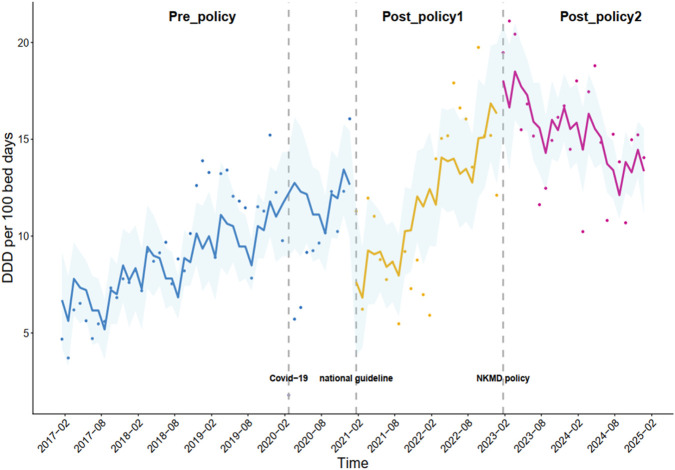
Monthly DDDs per 100 bed-days for all the oral PPIs combined.

Changes in the DDD per 100 bed-days for oral PPIs are shown in Table 2. Before the implementation of the policies, the consumption of oral PPIs exhibited a slightly increasing trend (β_0_ = 0.14, *p* < 0.001). The national guidelines (intervention_1_) resulted in a significant immediate reduction (β_1_ = −5.67, *p* = 0.005) in the consumption of oral PPIs. However, the trend for oral PPIs subsequently exhibited a significant upward slope (β_2_ = 0.26, *p* = 0.015) under the implementation of the national guidelines (intervention_1_). The consumption of oral PPIs showed a significant sustained linear decreasing trend (β_4_ = −0.58, *p* < 0.001) under the implementation of the NKMD policy (intervention_2_) (Figure 3).

**TABLE 2:** Interrupted time-series analyses of DDDs per 100 bed-days for oral PPIs.

Category	Newey–West coefficient	95% CI	*p*-value
Pre-intervention trend (β_0_)	0.14	0.06–0.21	**<0.001**
Change in the level for intervention_1_ (β_1_)	−5.67	−9.54 to −1.80	**0.005**
Change in the trend for intervention_1_ (β_2_)	0.26	0.05–0.47	**0.015**
Change in the level for intervention_2_ (β_3_)	0.80	−3.68 to 5.29	0.723
Change in the trend for intervention_2_ (β_4_)	−0.58	−0.79 to −0.37	**<0.001**
COVID-19 (β_5_)	−8.77	−11.45 to −6.09	**<0.001**
Constant	6.69	4.23 to 9.16	**<0.001**

Bold values indicate a *p*-value <0.05.

The national guidelines (intervention_1_) resulted in a significant immediate reduction in the consumption of oral PPIs. However, the trend for oral PPIs subsequently exhibited a significant upward slope under the implementation of the national guidelines (intervention_1_). The consumption of oral PPIs showed a significant sustained linear decreasing trend under the implementation of the NKMD policy (intervention_2_).

### Changes in the DDD per 100 bed-days for intravenous PPIs under the multifaceted stewardship

**FIGURE 4:**
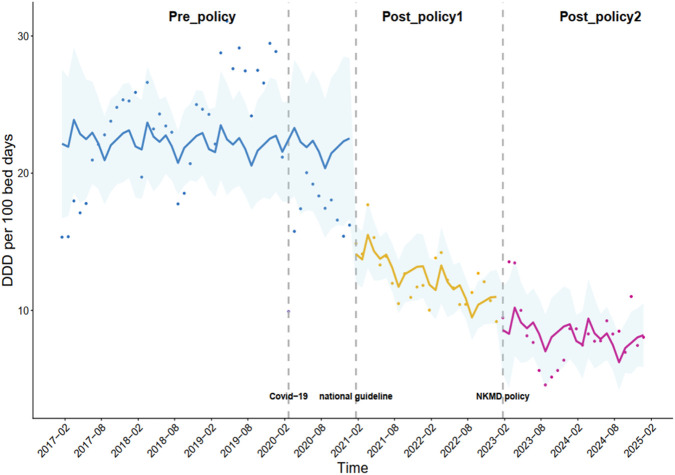
Monthly DDDs per 100 bed-days for all the intravenous PPIs combined.

Changes in the DDD per 100 bed-days for intravenous PPIs are shown in Table 3. A significant immediate reduction (β_1_ = −7.26, *p* = 0.020) in the consumption of intravenous PPIs was observed under the implementation of the national guidelines (intervention_1_). No significant difference was observed in the change in DDD per 100 bed-days for intravenous PPIs (*p*-values >0.05) under the implementation of the NKMD policy (Figure 4).

**TABLE 3:** Interrupted time-series analyses of the DDD per 100 bed-days for intravenous PPIs.

Category	Newey–West coefficient	95% CI	*p*-value
Pre-intervention trend (β_0_)	−0.02	−0.20 to 0.17	0.862
Change in the level for intervention_1_ (β_1_)	−7.26	−13.37 to −1.15	**0.020**
Change in the trend for intervention_1_ (β_2_)	−0.17	−0.39 to 0.05	0.131
Change in the level for intervention_2_ (β_3_)	−1.08	−7.67 to 7.52	0.591
Change in the trend for intervention_2_ (β_4_)	0.12	−0.07 to 0.32	0.223
COVID-19 (β_5_)	−11.42	−15.68 to −7.16	**<0.001**
Constant	22.14	16.73 to 27.56	**<0.001**

Bold values indicate a *p*-value <0.05.

A significant immediate reduction in the consumption of intravenous PPIs was observed under the implementation of the national guidelines (intervention_1_). No significant difference was observed in the change in the DDD per 100 bed-days for intravenous PPIs under the implementation of the NKMD policy (intervention_2_).

### Sensitivity analysis

Considering the time required for these policies to take effect, we set a 1-month lag to explore the impact of the multifaceted stewardship interventions on PPI consumption. The corresponding multi-intervention results are presented in Supplementary Tables S1–S3 and Supplementary Figures S1–S3 (see Supplement 3). The results of the sensitivity analysis were generally consistent with those of the main analysis, demonstrating the robustness and stability of the research findings.

### The appropriateness of prescription patterns of SUP

The demographic and clinical characteristics of the patients are presented in Table 4. No significant differences in terms of age, gender, occupational status, residence, comorbidities, and length of hospitalization were observed among the three groups (*p* > 0.05).

**TABLE 4:** Description of demographic and clinical characteristics of the study subjects.

Characteristic	Pre-policy	Post-policy_1_	Post-policy_2_	p*-*value*	p**-*value*	p***-*value*
Age (years)	57.83 ± 17.57	56.31 ± 16.51	54.50 ± 17.11	0.370	0.052	0.275
Gender				0.896	0.179	0.163
Male	102 (48.3%)	93 (47.7%)	111 (55.0%)	​	​	​
Female	109 (51.7%)	102 (52.3%)	91 (45.0%)	​	​	​
Occupational status				0.368	0.113	0.448
Employed	42 (19.9%)	46 (23.6%)	53 (26.5%)	​	​	​
Unemployed	169 (80.1%)	149 (76.4%)	147 (73.5%)	​	​	​
Residence				0.621	0.401	0.172
Rural	109 (51.9%)	106 (54.4%)	96 (47.8%)	​	​	​
Urban	101 (48.1%)	89 (45.6%)	105 (52.2%)	​	​	​
Comorbidities				0.301	0.994	0.281
No	119 (56.4%)	100 (51.3%)	114 (56.4%)	​	​	​
Yes	92 (43.6%)	95 (48.7%)	88 (43.6%)	​	​	​
Length of hospitalization (days)	14.77 ± 9.80	13.63 ± 9.33	13.15 ± 6.86	0.233	0.053	0.515

* indicates the comparison between post-policy_**1**_ and pre-policy; ** indicates the comparison between post-policy_2_ and pre-policy; and *** indicates the comparison between post-policy_2_ and post-policy_1_.

As shown in Table 5, the incidence of inappropriate indications for SUP use decreased significantly under the implementation of the NKMD policy (intervention_2_) (***p*** = 0.020). Our analysis included a total of 608 patients, corresponding to 2,717 patient-days of PPI use. These were distributed across the pre-policy and both post-policy periods. Compared to the pre-intervention period, the average percentage of patient-days with inappropriate SUP use decreased by 14.3% following the national guidelines (intervention_1_) and by 19.3% following the NKMD policy (intervention_2_). The average duration of inappropriate SUP use was calculated to be 5.80 ± 4.09 days in the pre-policy period, 4.77 ± 2.93 days in the post-policy_1_ period, and 3.19 ± 1.93 days in the post-policy_2_ period. A significant decreasing trend was observed across the three periods, with the sharpest decrease under the implementation of the NKMD policy (intervention_2_) (***p*** < 0.001). The rate of inappropriate timing of the first prophylactic dose also decreased significantly following the interventions (***p*** < 0.05).

**TABLE 5:** Impact of clinical pharmacist interventions on the rational use of stress ulcer prophylaxis for inpatients in the department of orthopedics.

Characteristic	Pre-policy (n = 211)	Post-policy_1_ (n = 195)	Post-policy_2_ (n = 202)	p*- *value*	p**- *value*	p***- *value*
The incidence of inappropriate indications for SUP use	46.4% (98/211)	37.4% (73/195)	35.1% (71/202)	0.066	**0.020**	0.684
The average percentage of patient-days with inappropriate SUP use	46.2% (568/1,230)	39.6% (348/878)	37.3% (227/609)	**0.003**	**<0.001**	0.358
The average duration of inappropriate SUP use	5.80 ± 4.09	4.77 ± 2.93	3.19 ± 1.93	0.069	**<0.001**	**<0.001**
The rate of inappropriate timing of the first prophylactic dose	72.5% (153/211)	37.4% (73/195)	21.3% (43/202)	**<0.001**	**<0.001**	**0.001**

Bold values indicate a *p*-value <0.05. * indicates the comparison between post-policy_**1**_ and pre-policy; ** indicates the comparison between post-policy_2_ and pre-policy; and *** indicates the comparison between post-policy_2_ and post-policy_1_.

## Discussion

### Unsustainable influence of the national guidelines on PPI use

Although the DDD per 100 bed-days for overall PPIs decreased by 12.93 (*p* = 0.009) under the national guidelines (Intervention_1_), no significant time trend in the overall PPI utilization was observed (*p* = 0.603). This study suggested that the implementation of the national guidelines (Intervention_1_) resulted in a significant immediate reduction, but no significant time trend in the overall PPI utilization was observed. The possible reasons are as follows: first, the translation of clinical guidelines into improved outcomes remains a challenge because of a variety of barriers (Cabana et al., 1999). The previous literature highlighted the difficulties in changing physicians’ prescribing behaviors according to the guidelines. A study demonstrated the ineffectiveness of the codes for PPI prescriptions in reducing inappropriate prescriptions, through which the prescribers were asked to reassess the use of PPIs and discontinue them if necessary (Nguyen et al., 2023). Efforts to combat the overprescribing of PPIs failed to significantly change clinical practice following the publication of a new treatment guideline in the United Kingdom (Abrahami et al., 2021). Interventions to enhance adherence to guidelines and promote the rational use of PPIs seem to have had an insubstantial influence on the overall prescribing rate in Denmark (Haastrup et al., 2014). The level or trend of PPI prescriptions is modestly affected by clinical guidelines in France (Yang et al., 2022). Second, it is difficult to maintain a long-term effect of PPI deprescribing with repeated time- and resource-consuming interventions because the interest and uptake of the guidelines might have receded over time (Colombet et al., 2009; Thompson et al., 2016). A previous study reported that the effect did not persist after a less resource-intensive intervention (Colombet et al., 2009). However, some other studies indicated that clinical guidelines are considered helpful for improving medical practice (Colombet et al., 2009; Jones et al., 2022; Ziegler et al., 2019).

### Effective intervention of the NKMD policy on PPI utilization

A significant decreasing trend (β_4_ = −0.46, *p* = 0.011) in the DDD per 100 bed-days for overall PPIs was observed under the implementation of the second batch of the NKMD policy catalog (intervention_2_). Previous literature demonstrated that the total DDDs for 10 NKMDs indicated sustained reductions over 19 months after multidimensional interventions under the implementation of the first batch of the NKMD policy catalog (Li et al., 2022b), which was consistent with our study. However, the findings of our study were inconsistent with those of previous studies indicating that the consumption of policy-related drugs decreased by 83.29% after the implementation of the first batch of the NKMD policy catalog (Wen et al., 2023).

Unlike the fact that there was no significant sustained downward trend in overall PPI consumption under the national guidelines (intervention_1_), the implementation of the NKMD policy (Intervention_2_) resulted in a significant downward trend (β_4_ = −0.46, *p* = 0.011) in overall PPI utilization, indicating that the effectiveness of the administrative policy was stronger than the guidelines. The strict enforcement of national guidelines, educational programs, prescription evaluation and audits, and administrative interventions were addressed as strong strategies throughout the periods of intervention_1_ and intervention_2_. Previous studies indicated that pharmacy-led interventions could significantly curb the increase in the consumption of PPIs and promote the rational use of PPIs (Chen et al., 2022; Hong et al., 2020; Leszcynski and Bente, 2023; Luo et al., 2018; Singh-Franco et al., 2020; Zhang et al., 2021). However, these studies focused on limited study periods and lacked long-term follow-up data on clinicians’ prescribing habits or patients’ clinical outcomes. Furthermore, the national educational initiatives were reported to be insufficient in curbing the overuse of PPIs in Australia (Bruno et al., 2020). PPIs included in the second batch of the NKMD policy catalog were marked with an eye-catching “Supervision” symbol on drug labels in the HIS, which served as a reminder for clinicians when prescribing PPIs. However, it is challenging to set rules for intercepting prescriptions with drug-related problems (DRPs) according to the clinical guidelines and drug descriptions in the HIS. As education can be a non-sustainable quality intervention and the indications for PPI utilization are difficult to remember and apply in a busy academic institute with high turnover (Masood et al., 2018), we hypothesized that timely warning alerts in the HIS were associated with the observed decreasing trend in PPI consumption. This finding motivated the development of pharmacist-driven computerized programs designed to ensure strict guideline adherence in PPI prescribing and to prevent inappropriate PPI prescriptions.

### Changes in the patterns of PPI type prescription under the multifaceted stewardship

The types of PPIs prescribed were influenced by the multifaceted stewardship interventions. A significant immediate reduction (β_1_ = −7.26, *p* = 0.020) in the consumption of intravenous PPIs was observed under the implementation of the national guidelines (intervention_1_). From 2015 to 2019, PPI use was continuously supervised by the health commissions of multiple provinces because of their overuse in hospitals, including Sichuan, Anhui, Qinghai, Jiangxi, Shanxi, Hunan, and Yunnan provinces. The nationwide campaign for promoting the rational use of PPIs from 2015 had a positive impact on hospitalized patients (Liu et al., 2022), which significantly reduced PPI consumption and improved the appropriateness of PPI prescribing (Luo et al., 2018). Medical institutions were required to develop intervention and supervision measures to further standardize PPI prescribing. A longstanding challenge in curbing PPI overuse lies mainly in the absence of clear, unified evaluation criteria. Therefore, the national guidelines, serving as the guiding principles for PPI use, have established the basis for standardizing clinical prescribing practices. Our previous studies demonstrated that SUP prescribed for patients admitted to surgical wards with a low risk of stress-related mucosal disease (SRMD) was a major contributor to inappropriate PPI use in our hospital (Li et al., 2022a). In addition, intravenous PPIs accounted for 95.3% of the prescriptions among inpatients receiving acid suppression therapy (AST), as described in the previous study. Early prophylactic administration of acid-suppressing agents is effective in reducing the incidence of stress ulcers. According to the national guidelines, oral PPIs are recommended as the first-line acid-suppressing therapy. Following the implementation of clinical pharmacist-led interventions, our hospital demonstrated a marked improvement in the appropriate use of intravenous PPIs among inpatients (Li et al., 2019). We hypothesized that the decrease in intravenous PPI use was attributable to the standardization of SUP, achieved through pharmacist-led interventions in perioperative prophylaxis practices following the national guidelines.

Before the implementation of the interventions (between January 2017 and December 2020), the consumption of oral PPIs exhibited a slightly increasing trend (β_0_ = 0.14, *p* < 0.001). A significant immediate reduction (β_1_ = −5.67, *p* = 0.005) in the consumption of oral PPIs was observed under the implementation of the national guidelines (intervention_1_). However, the trend for oral PPI use subsequently exhibited a significant upward slope (β_2_ = 0.26, *p* = 0.015) under the implementation of the national guidelines (intervention_1_). A study demonstrated that PPI use decreased in the first 6 months after guideline implementation but subsequently returned to baseline (Thompson et al., 2016), which was consistent with our study. The consumption of oral PPIs showed a significant sustained linear decreasing trend (β_4_ = −0.58, *p* < 0.001) under the NKMD policy implementation (Intervention_2_). The reasons for the decreasing trend in oral PPIs might be multifactorial. Oral PPIs are associated with more frequent inappropriate prophylactic use of PPIs during the perioperative period in recent years (Liu et al., 2024). As recommended in the guidelines and NKMD policy, clinicians were educated to carefully evaluate the indications and duration of PPI treatment in clinical practice to prevent the medical burden of patients and the occurrence of adverse drug reactions (ADRs) (Chen et al., 2022). Our finding of the significant decreasing trend in oral PPI consumption might be explained by the possibility that unnecessary prescriptions of oral PPIs were significantly reduced due to the strict restrictions of the second batch of the NKMD policy catalog (intervention_2_).

### The appropriateness of prescription patterns of SUP

The main purpose of implementing policies was to improve the rationality of drug use, matching a drug to a specific patient—right drug for the right patient at the right time and at the right dose (Mela et al., 2023). This study provided evidence that multifaceted stewardship interventions were effective in promoting the rational use of SUP. Prior to intervention, 46.4% of the 211 patients were deemed with inappropriate indications for SUP use based on the national guidelines in this study, which was lower than that reported in previous studies highlighting that 68.9% of the 274 patients received PPI prescriptions without appropriate indications (Alam et al., 2025). The average percentage of patient-days with inappropriate SUP use was 46.2% during the pre-intervention period, which was higher than that reported in the previous study (41.2%) (Buckley et al., 2015). Pharmacist interventions resulted in marked reductions in the incidence of inappropriate indications for SUP use (intervention_2_), the average duration of inappropriate SUP use (intervention_2_), and the average percentage of patient-days with inappropriate SUP use (intervention_1_ and intervention_2_), which was consistent with the previous study (Masood et al., 2018; Buckley et al., 2015). A significant reduction in the incidence of inappropriate indications for SUP use (*p* = 0.020) and the average duration of inappropriate SUP use (*p* < 0.001) was observed under the implementation of the NKMD policy (intervention_2_), whereas no significant differences were observed under the implementation of the national guidelines (intervention_1_). These results align with ITS analysis suggesting that the effectiveness of the administrative policy (Intervention_2_) was stronger than that of the guidelines (intervention_1_).

## Practical implications

For the healthcare financing system, optimal, evidence-based planning of the therapeutic process reduces losses arising from the haphazard and ineffective use of expensive therapies (Mela et al., 2023). Measures to consistently reduce unnecessary PPI prescriptions must be explored imperatively. The previous literature demonstrates that the clinical guidelines may lead to changes in prescribing behaviors if supported by computerized reminders, which would allow real-time monitoring of ordering practices and quality improvement efforts (Colombet et al., 2009; Monteiro et al., 2019). In China, the prospective prescription review system is a real-time prescription monitoring system that requires clinical pharmacists to establish rules for intercepting prescriptions with DRPs according to clinical guidelines, medical insurance policy, and drug descriptions (Fan et al., 2023; Xie et al., 2022; Yu et al., 2021). Since the prescription review was required in medical institutions by the National Health Commission in July 2018, approximately 42.7% of hospitals have successively carried out a prospective prescription review system (Fan et al., 2023). The prospective prescription review system is worthy of long-term implementation to further promote the rational use of PPIs and is associated with reduced spending toward PPIs and improved clinical outcomes (Fan et al., 2023).

### Strengths and limitations

The study addressed a considerable gap in the literature, being the most recent and comprehensive investigation of the effects of the multifaceted stewardship intervention on PPI utilization in a large tertiary hospital. The research results might be valuable references for the follow-up policy promotion and improvement. This study had several limitations. First, our observation was limited to a single hospital; thus, the results may not fully represent the overall implementation effect of the multifaceted stewardship intervention in China, and caution should be exercised when generalizing the findings. Despite this limitation, a single-group ITS study can identify policy effects through self-comparisons before and after the intervention. Our results demonstrated a representative evaluation of the multi-approach stewardship interventions on PPI utilization in pilot cities. The second limitation was that fluctuations in the PPI consumption due to strict restrictions on mobility of the population during the COVID-19 pandemic were associated with confounding factors, so there may be some deviation in the results. A sharp decrease in the DDD per 100 bed-days for PPIs was observed around February 2020, which was consistent with the lockdown in China during the outbreak of the COVID-19 pandemic (Zhang et al., 2024). Third, the rationality of clinical medication use cannot be judged simply by the decreasing rate of drug consumption. Clinical practice guidelines were published to ensure that doctors’ prescribing behaviors were appropriate to clinical needs. PPIs should only be used when the patient’s condition aligns with the corresponding indications, which could decrease not only unjustifiable medical expenses but also adverse drug reactions. Further quantitative studies on the rational use of PPIs are warranted to explore the effects of the multifaceted stewardship interventions.

## Conclusion

The implementation of the national guidelines led to a significant immediate reduction, but no significant time trend in the overall PPI utilization was observed. Our findings highlight the impact of the NKMD policy on the sustained downward trend in overall and oral PPI consumption. The prospective prescription review system may be effective in promoting the long-term rational use of PPIs in clinical practice.

## Data Availability

The original contributions presented in the study are included in the article/Supplementary Material; further inquiries can be directed to the corresponding authors.
