# Supplementary material for: Evaluating a multifaceted stewardship intervention on proton pump inhibitor utilization: an interrupted time-series analysis of prescribing patterns in a northwest Chinese hospital
Source: Front Pharmacol. 2026 Feb 10;17:1700146. doi: 10.3389/fphar.2026.1700146 (PMC12929134; doi:10.3389/fphar.2026.1700146)
Supplement: Supplementary file 1 [file Supplementaryfile1.docx]

**Supplement 1**

**Intervention measures developed through the implementation of the national guideline and the NKMDs policy at Xi’an People’s Hospital (Xi’an Fourth Hospital)**

1. *the establishment of a management group*

The guidelines for the clinical application of PPIs and the second batch of catalogue of NKMDs were published at the study hospital in January 2021 and January 2023, respectively. A scientific management system supported by the Pharmaceutical Management Professional Committee (PMPC) was established to prevent inappropriate prescription of PPIs in the hospital in January 2021. Regimens for the management and supervision of the rational application of PPIs were also formulated. A management leading group of the second batch of the NKMDs policy was set up by the medical department and department of pharmacy jointly in January 2023, which was attached to the PMPC of the hospital. This management leading group is responsible for supervising the implementation of the national guideline and the NKMDs policy, which was composed of administrative staff, pharmacists, and multidisciplinary clinicians in the hospital. With the assistance of the medical department, the management responsibility was clarified. The department of pharmacy was responsible for training doctors on the rational use of PPIs, providing consultation for doctors and standardizing their prescription behaviors. Clinical pharmacists were responsible for prescription review, data collection and feedback.

*2. educational programs*

The Guiding Principles for Clinical Application of PPIs and Prescription Management Measures were released to regulate prescribing patterns of all clinicians. Clinicians were mandated to prescribe PPIs in a guideline-based manner after completing training on the rational use of PPIs in the lecture hall or clinical departments. All clinicians were required to attend the training at least once. Clinical pharmacists participated in ward rounds and advised doctors to modify unreasonable prescriptions with PPIs.

*3. prescription evaluation and audits*

Retrospective evaluation of PPI prescriptions was performed monthly by clinical pharmacists. Regular monitoring reports were issued by department of pharmacy monthly, including unreasonable prescriptions of PPIs and related early warning information. The hospital intranet provided all medical staff with access to this report.

*4. cooperation with information department*

With the support of the information department, each PPI product was marked with an eye-catching ‘Supervision’symbol in front of the drug names in hospital information system (HIS) if necessary, indicating that this drug was monitored as NKMDs. With the training provided by clinical pharmacists, clinicians were reminded to pay attention to the indications while prescribing. These could greatly restrict the prescription of NKMDs and influence the prescribing pattern of PPIs.

*5. administrative interventions*

Clinicians who prescribed PPIs inappropriately were provided face-to-face guidance by clinical pharmacists to help them understand problems. When an error occurred for the first time, the clinician was given a warning and was required to rectify it. If the clinician refused to rectify after 3 consecutive warnings, administrative penalties were imposed.
