# Supplementary material for: Evaluating a multifaceted stewardship intervention on proton pump inhibitor utilization: an interrupted time-series analysis of prescribing patterns in a northwest Chinese hospital
Source: Front Pharmacol. 2026 Feb 10;17:1700146. doi: 10.3389/fphar.2026.1700146 (PMC12929134; doi:10.3389/fphar.2026.1700146)
Supplement: Supplementary file 2 [file Supplementaryfile3.docx]

**STABLE 1** Interrupted Time Series analyses for DDD per 100 bed days for overall PPIs

(1-month lag period)

| Categories | Newey-West Coefficient | 95%CI | *p*-value |
| --- | --- | --- | --- |
| Pre-intervention trend (β_0_) | 0.10 | -0.12-0.33 | 0.374 |
| Change in level for intervention1 (β_1_) | -13.32 | -22.30- -4.33 | **0.004** |
| Change in trend for intervention1 (β_2_) | 0.20 | -0.12-0.53 | 0.217 |
| Change in level for intervention2 (β_3_) | -2.28 | -9.46-4.89 | 0.527 |
| Change in trend for intervention2 (β_4_) | -0.52 | -0.90 - -0.14 | **0.008** |
| COVID-19 (β_5_) | -20.01 | -26.08- -13.95 | **<0.001** |
| Constant | 27.54 | 20.27-34.82 | **<0.001** |

Bold values indicated a *p*-value<0.05.

**STABLE 2** Interrupted Time Series analyses for DDD per 100 bed days for oral PPIs

(1-month lag period)

| Categories | Newey-West Coefficient | 95%CI | *p*-value |
| --- | --- | --- | --- |
| Pre-intervention trend (β_0_) | 0.13 | 0.08-0.19 | **<0.001** |
| Change in level for intervention1 (β_1_) | -6.19 | -9.93- -3.13 | **0.001** |
| Change in trend for intervention1 (β_2_) | 0.34 | 0.16-0.51 | **<0.001** |
| Change in level for intervention2 (β_3_) | -0.97 | -3.64-2.97 | 0.583 |
| Change in trend for intervention2 (β_4_) | -0.63 | -0.87- -0.40 | **<0.001** |
| COVID-19 (β_5_) | -8.88 | -11.34- -6.42 | **<0.001** |
| Constant | 6.09 | 3.58-8.60 | **<0.001** |

Bold values indicated a *p*-value<0.05.

**STABLE 3** Interrupted Time Series analyses for DDD per 100 bed days for intravenous PPIs (1-month lag period)

| Categories | Newey-West Coefficient | 95%CI | *p*-value |
| --- | --- | --- | --- |
| Pre-intervention trend (β_0_) | -0.03 | -0.21-0.15 | 0.721 |
| Change in level for intervention1 (β_1_) | -7.13 | -13.31- -0.09 | **0.024** |
| Change in trend for intervention1 (β_2_) | -0.137 | -0.34-0.067 | 0.184 |
| Change in level for intervention2 (β_3_) | -1.32 | -5.28- 2.65 | 0.511 |
| Change in trend for intervention2 (β_4_) | 0.113 | -0.10-0.33 | 0.299 |
| COVID-19 (β_5_) | -11.13 | -15.31- 6.96 | **<0.001** |
| Constant | 21.45 | 15.98-26.92 | **<0.001** |

Bold values indicated a *p*-value<0.05.


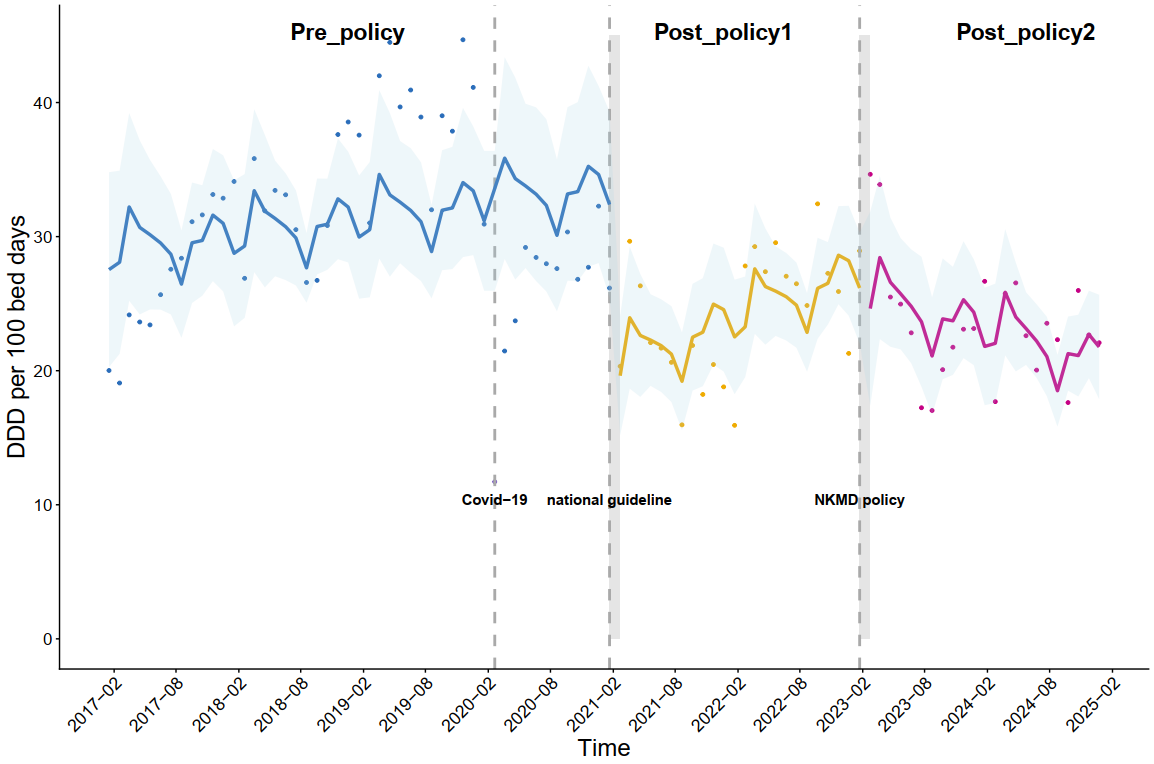


Figure S1 Monthly DDD per 100 bed days of all the PPIs combined (1-month lag period)


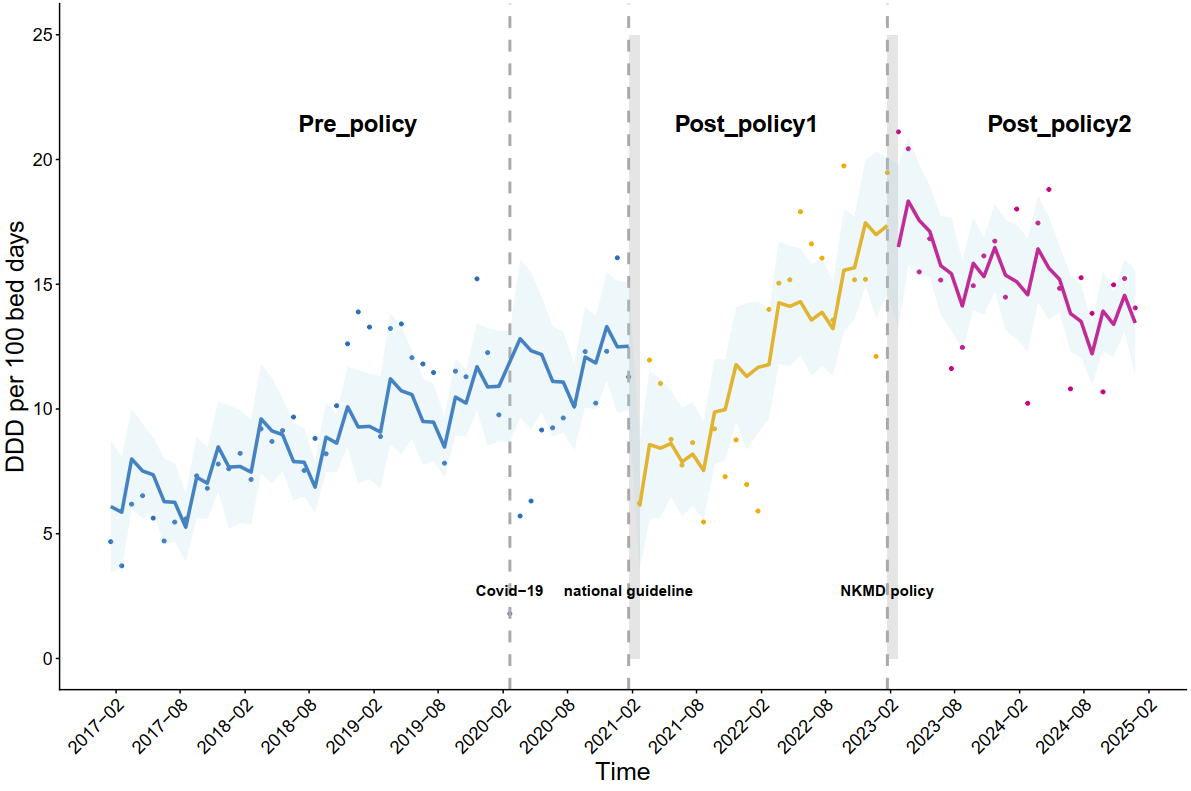


Figure S2 Monthly DDD per 100 bed days of all the oral PPIs combined (1-month lag period)


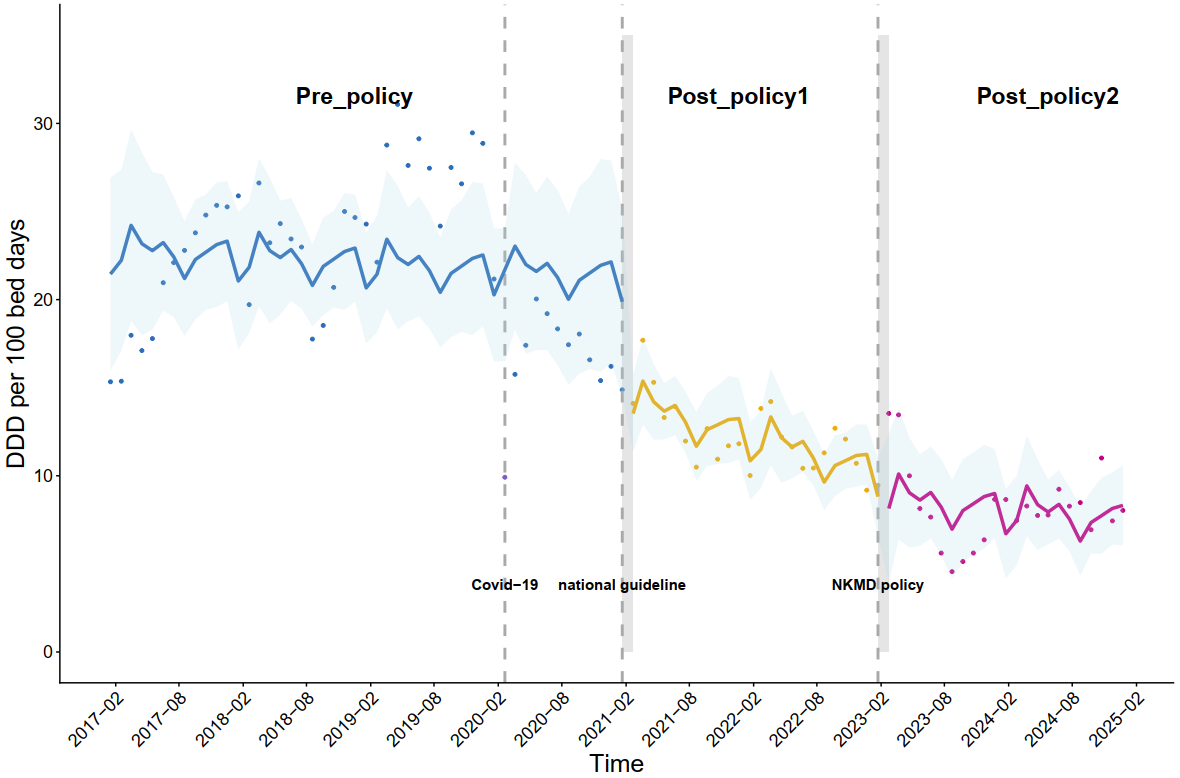


Figure S3 Monthly DDD per 100 bed days of all the intravenous PPIs combined (1-month lag period)
