# Supplementary material for: Evaluating a multifaceted stewardship intervention on proton pump inhibitor utilization: an interrupted time-series analysis of prescribing patterns in a northwest Chinese hospital
Source: Front Pharmacol. 2026 Feb 10;17:1700146. doi: 10.3389/fphar.2026.1700146 (PMC12929134; doi:10.3389/fphar.2026.1700146)
Supplement: Supplementary file 3 [file Supplementaryfile2.docx]

Statistical Appendix

1. Model Fitting and Diagnosis

1.1 Overall PPIs

① monthly trends assessment


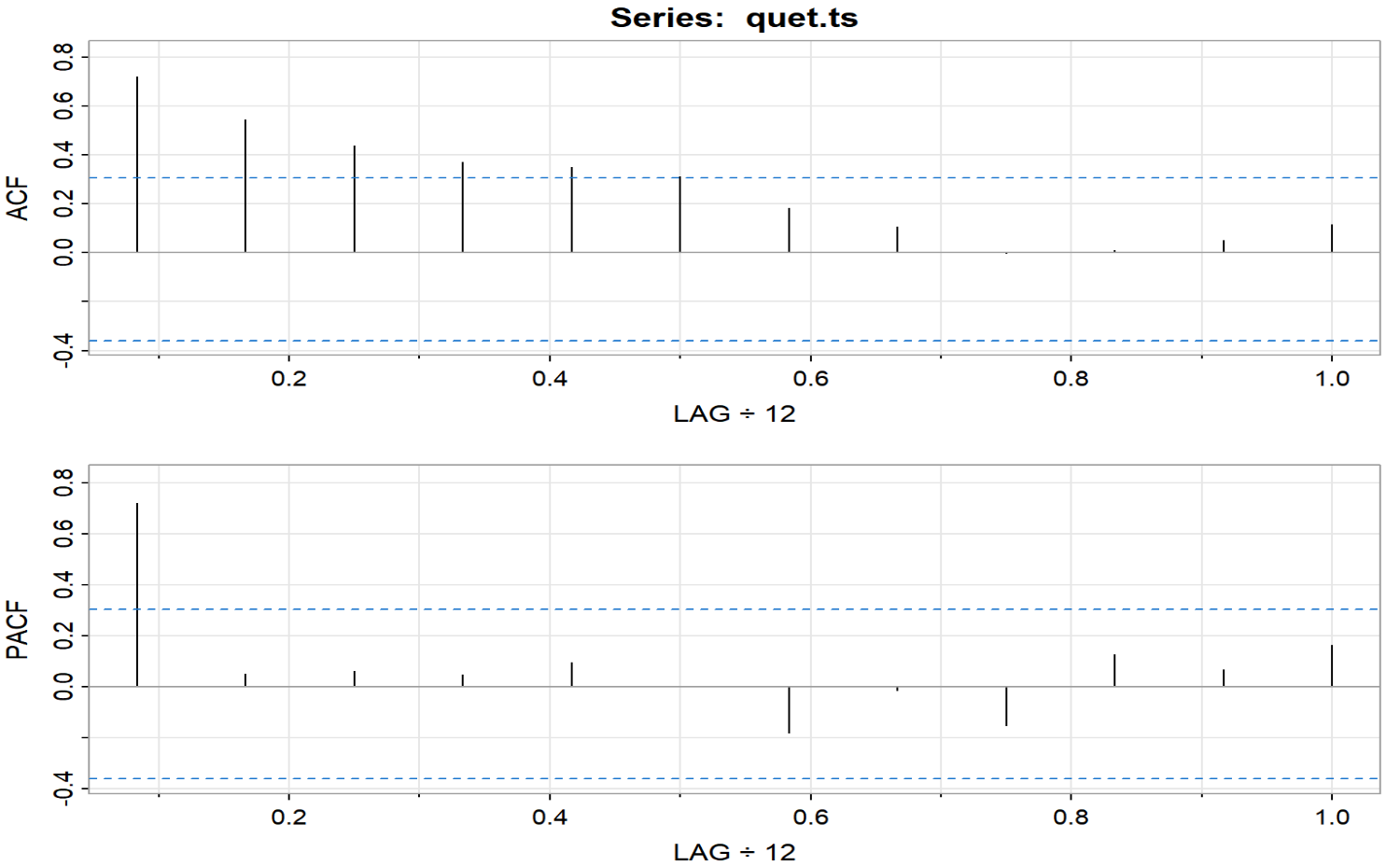


② model fitting results

Residual standard error: 5.476 on 78 degrees of freedom

Multiple R-squared: 0.4772, Adjusted R-squared: 0.3632

③ model diagnosis and residual autocorrection test


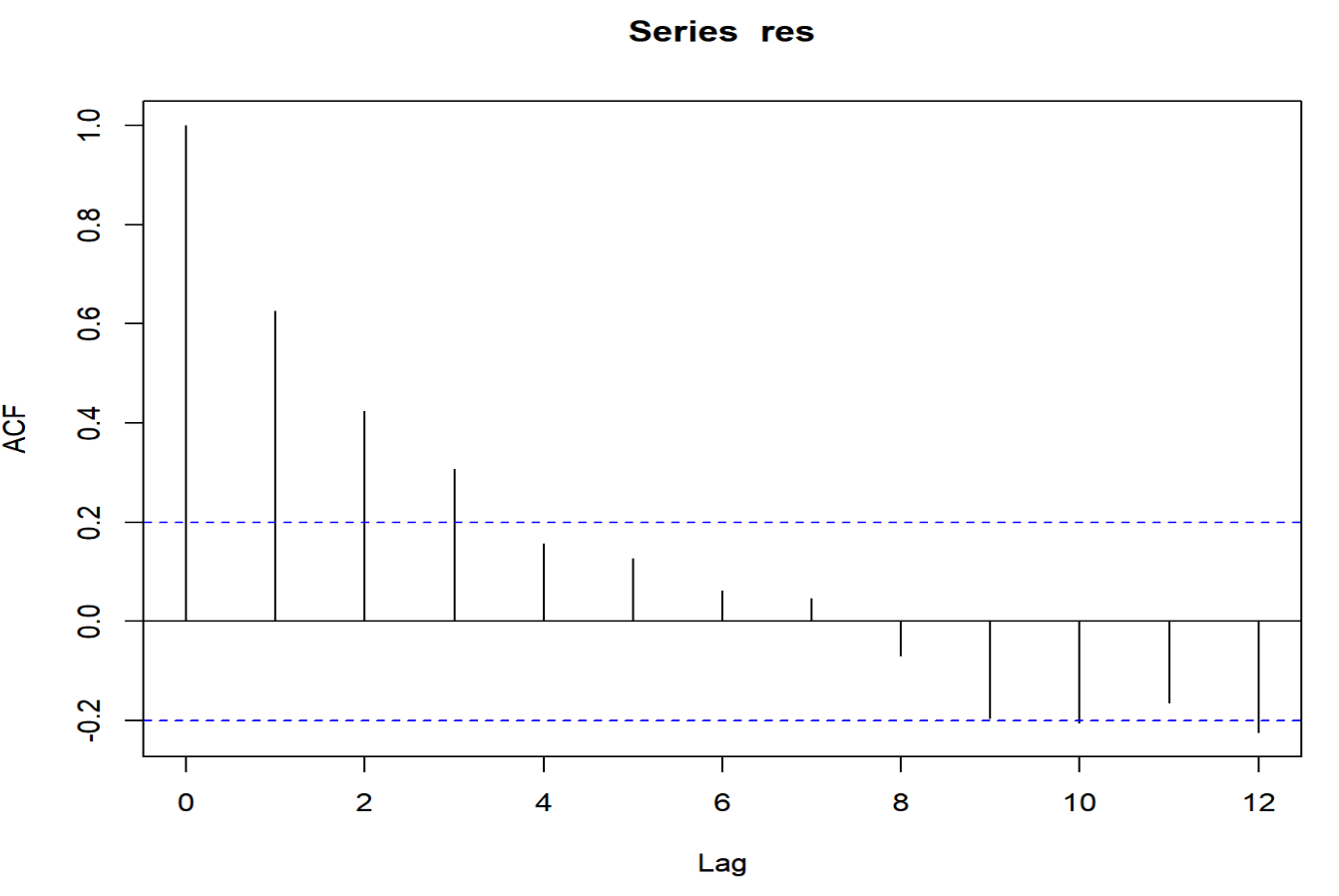


④ Newey-West method adjustment coefficient

**TABLE 1** Interrupted Time Series analyses for DDD per 100 bed days for overall PPIs

| Categories | Newey-West Coefficient | 95%CI | *p*-value |
| --- | --- | --- | --- |
| Pre-intervention trend (β_0_) | 0.12 | -0.13-0.37 | 0.331 |
| Change in level for intervention1 (β_1_) | -12.93 | -22.54- -3.32 | **0.009** |
| Change in trend for intervention1 (β_2_) | 0.09 | -0.26-0.45 | 0.603 |
| Change in level for intervention2 (β_3_) | -0.28 | -8.04-7.48 | 0.943 |
| Change in trend for intervention2 (β_4_) | -0.46 | -0.82- -0.11 | **0.011** |
| COVID-19 (β_5_) | -20.19 | -26.62- -13.75 | **<0.001** |
| Constant | 28.84 | 21.51-36.16 | **<0.001** |

1.2 oral PPIs utilization

① monthly trends assessment


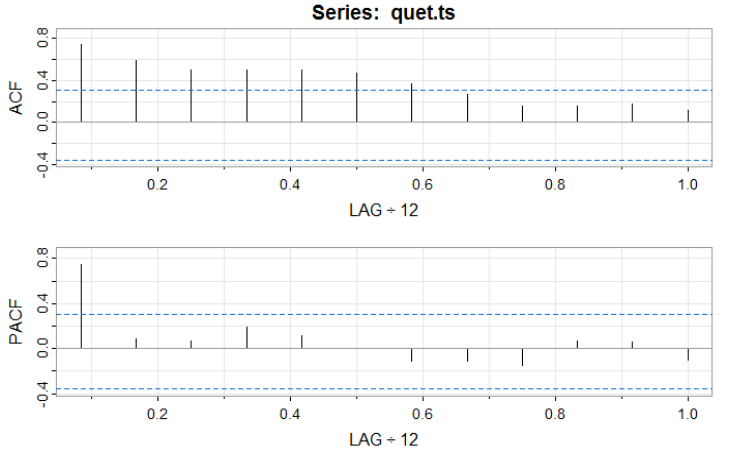


② model fitting results

Residual standard error: 2.634 on 78 degrees of freedom

Multiple R-squared: 0.6763, Adjusted R-squared: 0.6057

③ model diagnosis and residual autocorrection test


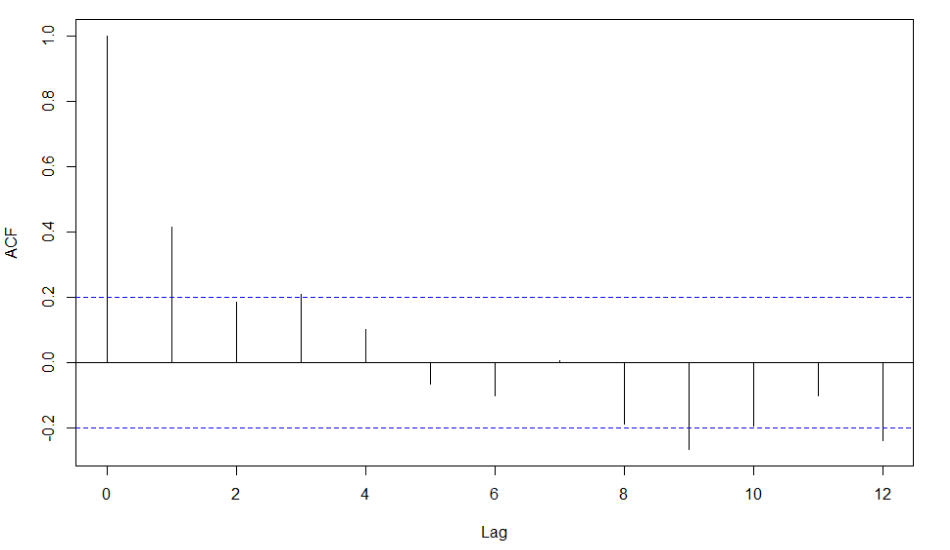


④ Newey-West method adjustment coefficient

**TABLE 2** Interrupted Time Series analyses for DDD per 100 bed days for oral PPIs

| Categories | Newey-West Coefficient | 95%CI | *p*-value |
| --- | --- | --- | --- |
| Pre-intervention trend (β_0_) | 0.14 | 0.06-0.21 | **<0.001** |
| Change in level for intervention1 (β_1_) | -5.67 | -9.54- -1.80 | **0.005** |
| Change in trend for intervention1 (β_2_) | 0.26 | 0.05-0.47 | **0.015** |
| Change in level for intervention2 (β_3_) | 0.80 | -3.68- 5.29 | 0.723 |
| Change in trend for intervention2 (β_4_) | -0.58 | -0.79- -0.37 | **<0.001** |
| COVID-19 (β_5_) | -8.77 | -11.45- -6.09 | **<0.001** |
| Constant | 6.69 | 4.23-9.16 | **<0.001** |

1.3 Intravenous PPIs

① monthly trends assessment


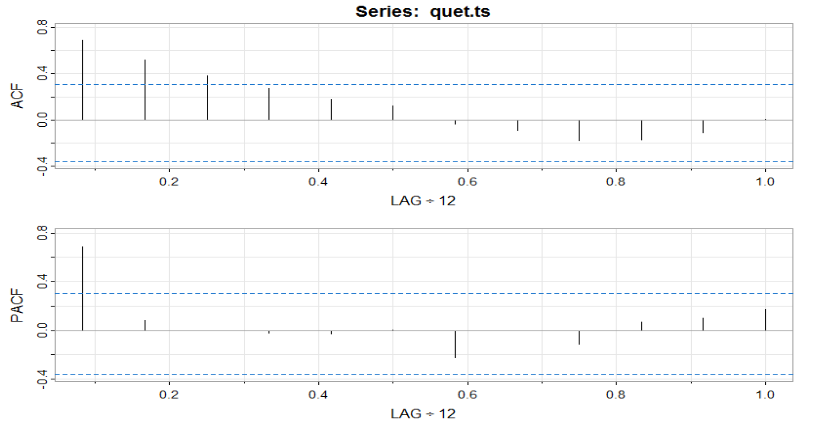


② model fitting results

Residual standard error: 3.619 on 78 degrees of freedom

Multiple R-squared: 0.7838, Adjusted R-squared: 0.7367

③ model diagnosis and residual autocorrection test


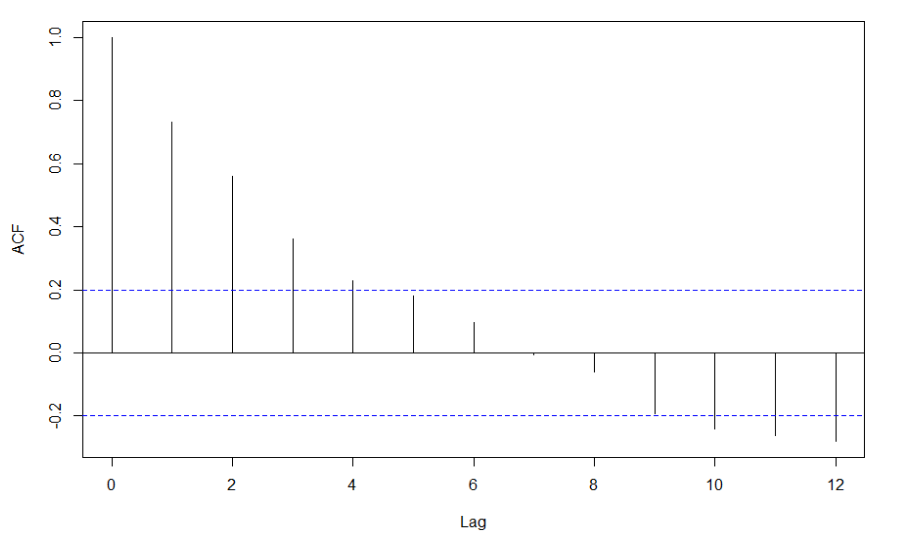


④ Newey-West method adjustment coefficient

**TABLE 3** Interrupted Time Series analyses for DDD per 100 bed days for intravenous PPIs

| Categories | Newey-West Coefficient | 95%CI | *p*-value |
| --- | --- | --- | --- |
| Pre-intervention trend (β_0_) | -0.02 | -0.20-0.17 | 0.862 |
| Change in level for intervention1 (β_1_) | -7.26 | -13.37- -1.15 | **0.020** |
| Change in trend for intervention1 (β_2_) | -0.17 | -0.39-0.05 | 0.131 |
| Change in level for intervention2 (β_3_) | -1.08 | -7.67-7.52 | 0.591 |
| Change in trend for intervention2 (β_4_) | 0.12 | -0.07- 0.32 | 0.223 |
| COVID-19 (β_5_) | -11.42 | -15.68- -7.16 | **<0.001** |
| Constant | 22.14 | 16.73-27.56 | **<0.001** |
